# Readiness to Embrace Artificial Intelligence Among Medical Students in Saudi Arabia: A National Survey

**DOI:** 10.3390/healthcare12242504

**Published:** 2024-12-11

**Authors:** Abeer Al Shahrani, Norah Alhumaidan, Zeena AlHindawi, Abdullah Althobaiti, Khalid Aloufi, Rasil Almughamisi, Ahad Aldalbahi

**Affiliations:** 1 Family and Community Medicine Department, College of Medicine, Princess Nourah bint Abdulrahman University, Riyadh 11671, Saudi Arabia; 2 College of Medicine, Princess Nourah bint Abdulrahman University, Riyadh 11671, Saudi Arabia; noura_alhumaidan@outlook.com (N.A.); alizaina74@gmail.com (Z.A.); 3 College of Medicine, Taif University, Taif 21944, Saudi Arabia; abdullahabalmansori@gmail.com; 4 College of Medicine, Northern Border University, Arar 73213, Saudi Arabia; alwfy3461@gmail.com; 5 College of Medicine, Taibah University, Al Madinah Al Munawwarah 42353, Saudi Arabia; tu4150406@taibahu.edu.sa; 6 College of Medicine, King Faisal University, Hofuf 31982, Saudi Arabia; ahad.kfu@gmail.com

**Keywords:** artificial intelligence, medical education, curriculum, medical students, Saudi Arabia

## Abstract

Background/Objectives: Artificial intelligence (AI) is rapidly reshaping healthcare, offering transformative potential for diagnostics, treatment, and patient management. Despite its growing significance, there is limited integration of AI education in medical curricula, raising concerns about the readiness of future healthcare professionals to utilize AI technologies. This study aims to evaluate the readiness of medical students in Saudi Arabia to embrace AI and to assess the current state of AI education, AI Application use, and future perspectives for medical students. Methods: a cross-sectional design was employed. It involved medical students from various regions of Saudi Arabia. Data were collected using an anonymous, online, structured, and validated tool from previous studies. The survey included sociodemographic information, details on AI education, the usage of AI applications, intended specialties, and a Medical Artificial Intelligence Readiness Scale for Medical Students (MAIRS-MS). The data were extracted and revised in an Excel sheet. Statistical analysis was conducted using the IBM SPSS computer program with appropriate statistical tests. Results: This study enrolled 572 medical students, with a mean age of 21.93 years. Most students were Saudi (99.0%), and 43.7% lived in the western region of Saudi Arabia. Most students attended a government medical college (97.41%), and 64.3% of students were in their clinical years. Only 14.5% of the students had received formal AI education, while 34.3% had participated in extracurricular AI training. The mean (SD) MAIRS-MS score was 68.39 (18.3), with higher scores associated with female students, those from the central region, and those with advanced English and computer technology skills (*p* < 0.001). Conclusions: there is limited AI education and moderate AI readiness among medical students in Saudi colleges, with significant variability in terms of gender, region, and educational background. These findings underscore the need to integrate AI education into medical curricula to better prepare future physicians for AI-enabled healthcare systems.

## 1. Introduction

Artificial intelligence (AI) refers to a broad range of technologies designed to perform tasks that typically require human intelligence, such as decision-making, pattern recognition, and problem-solving, without direct human intervention [1]. With its potential to enhance evidence-based clinical decision-making and contribute to value-based care, AI is rapidly transforming healthcare [2]. As the availability of healthcare data and investments in AI by technology companies continue to rise, AI applications in medicine have become increasingly useful. For example, AI systems now assist physicians in fields such as radiology, pathology, and precision oncology [3]. Moreover, AI plays a critical role in patient care through innovations in remote patient monitoring, telemedicine, and virtual support systems [4].

Beyond clinical practice, AI is also making inroads into medical education. AI-based platforms are being used in case-based online learning, virtual standardized patient systems for history taking, and other educational tools designed to improve medical training [5,6]. Internationally, leading academic institutions in the USA and Canada have begun integrating AI-focused modules/courses that address key topics such as machine learning, data analytics, and AI ethics into undergraduate/postgraduate medical curricula [7]. These initiatives aim to prepare healthcare professionals for a digital future where AI is expected to play a significant role in clinical decision-making and healthcare delivery [8].

Despite these advancements, the implementation of AI education in medical schools remains inconsistent globally. Key gaps include the absence of standardized curricula [9], insufficient practical application of AI tools [10], limited availability of faculty with AI expertise [11], limited students’ knowledge and skills [12], and variability in students’ attitudes and levels of preparedness [13,14]. Furthermore, critical aspects such as the ethical implications and policy considerations of AI are often insufficiently addressed in current training programs [15,16]. These gaps highlight the necessity for the development of comprehensive and standardized educational frameworks to adequately prepare future physicians for the integration of AI into healthcare systems [17].

In the context of Saudi Arabia, these global gaps are compounded by several local challenges. The country’s Vision 2030 initiative emphasizes the integration of advanced technologies, including AI, to transform healthcare and establish a technology-driven economy [18]. However, the readiness of medical students to meet these demands has not been comprehensively investigated. Evaluating their preparedness is crucial to ensuring that the healthcare workforce aligns with national strategic objectives [19,20]. Furthermore, existing studies in Saudi Arabia provide only limited insights into medical students’ readiness and the current state of AI education in medical institutions [21,22]. To date, Saudi medical colleges have not fully incorporated AI into their curricula, and without a clear understanding of the gaps in knowledge, skills, and attitudes among medical students, it remains challenging to design and implement effective curriculum reforms [23].

Therefore, this study aims to evaluate the preparedness of medical students in Saudi Arabia regarding AI technologies and their applications. Moreover, this study aims to assess the current state of AI education in Saudi medical colleges and AI use and future perspectives for medical students.

## 2. Materials and Methods

The methodology was structured into distinct phases to ensure rigor and reliability. The initial phase involved defining the target population, calculating the required sample size using established statistical tools, and specifying inclusion and exclusion criteria. This was followed by the instrument development phase, during which the questionnaire was designed, translated, and validated through a pilot test. The data collection phase utilized a web-based survey distributed via social media platforms to recruit participants and collect responses. These phases are outlined below.

### 2.1. Study Design, Population, and Sample Size

This descriptive, cross-sectional study was conducted in Saudi Arabia. It included medical students who agreed to participate and completed the questionnaire. We excluded non-medical students, pre-medical/foundation-year students, and participants not living in Saudi Arabia. The sample size for this study was calculated using a Raosoft web-based calculator [24]. The total population size used for the calculation represents the number of medical students enrolled at Saudi medical colleges from 2022 to 2023 [25], using a 95% confidence interval with a 5% margin of error; a population size of 20,000 was estimated by Raosoft for an unknown population with a 50% response distribution. A total of 377 participants were required. As the study was conducted using a web-based survey with recruitment via social media, an estimated dropout rate of 40% was factored in. Therefore, to compensate for dropouts, we continued recruiting participants until 527 was reached.

### 2.2. Questionnaire Development and Data Collection

The instrument for the study was an online, self-administered, closed-ended questionnaire created using Google Forms. The questionnaire was distributed to individuals via social media platforms, including WhatsApp, Facebook, and Twitter, in Saudi Arabia. A snowball sampling technique was employed to recruit participants. The questionnaire was initially constructed in English and was formulated based on a previously validated questionnaire from similar studies [26,27,28]. Then, it was translated into Arabic. The translation into Arabic followed a forward and backward translation approach to ensure accuracy and linguistic consistency. A pilot test was then conducted with ten bilingual participants to assess question clarity and determine the estimated time required for completion.

The first section of the questionnaire consisted of six questions that covered sociodemographic data, which included age, gender, nationality, area of residence, type of medical college, and medical school year. The second section consisted of eight questions about artificial intelligence (AI) education. This included proficiency in English, proficiency in computer technology, curricular studies of ethics, data science, AI, extracurricular AI training, and common methods of AI training. The third section assessed the current usage of AI applications and future perspectives through five questions.

The last section included a scale, the Medical Artificial Intelligence Readiness Scale for Medical Students (MAIRS-MS), to assess the readiness of medical students for using AI [28]. It contained twenty-two phrases, with a five-point Likert scale (1 = Strongly Disagree, 2 = Disagree, 3 = Neutral, 4 = Agree, 5 = Strongly Agree), that were translated as well.

### 2.3. Statistical Analysis

The data were extracted and revised in an Excel sheet. Statistical analysis was conducted using the IBM SPSS computer program (version 26.0; Armonk, NY, USA). Categorical variables were presented as numbers and percentages. Continuous, nonnormally distributed variables were described using their mean, standard deviation (SD), median, and interquartile range (IQR). The score for each factor ranged from 0 to 5 (Likert-type rating scale). The total score was the sum of all factors (out of 110 points). The associations between the four factors and the total score with the independent variables were determined via the Mann–Whitney and Kruskal–Wallis tests. Linear regression was used to investigate the confounding variable. *p* Values less than 0.05 were considered statistically significant.

## 3. Results

This section presents a detailed analysis of the study’s findings, emphasizing the sociodemographic characteristics of participants, levels of English and computer skills proficiency, AI education and training, utilization of AI applications, intended specialty and the impact of AI advancement, readiness for AI integration in healthcare via MAIRS-MS, and factors influencing these aspects, as outlined below.

### 3.1. Summary of the Survey Respondents

It included 572 medical students, with a mean age of 21.93 years (SD = 1.844). Most participants were Saudi nationals residing in the Western region and attending government medical colleges. A total of 64.3% of students were in their clinical years (third to fifth year of the medical program) compared to 21.5% who were in the preclinical years. Further details of the study population are presented in Table 1.

### 3.2. Proficiency Levels, Artificial Intelligence Education and Training

Approximately half of the medical students reported intermediate proficiency in English and computer technology. Regarding their curricular education, most participants had received ethics and data science education; however, only 14.5% had received formal AI education within their curriculum. Additionally, 34.3% of students had participated in extracurricular AI training, with 56.6% (n = 111) of these students acquiring their training through self-study, as detailed in Table 2.

### 3.3. Status of Artificial Intelligence Applications Use

Most students used AI applications (93.2%, n = 533), and ChatGPT (60.4%, n = 322), Grammarly (51%, n = 272), and Poe (40.3%, n = 215) were the most commonly used applications. Among respondents who selected multiple objectives for utilizing AI applications, the most reported objectives were querying medical knowledge (68.2%, n = 388) and medical research (55.2%, n = 314), as shown in Table 3.

### 3.4. AI Dvancment and Its Impact on Intended Speciality

With respect to AI advancement and specialty choice, 33.7% (n = 193) of the students had not decided on their future specialty yet. However, among those who responded to this question (66.26%, n = 379), 18.7% intended to choose surgery, followed by internal medicine, family medicine, and ophthalmology, respectively. The remaining 0.2–4.7% of the students chose other specialties and subspecialties. Additionally, 28.5% of the students responded that AI advancement in healthcare will impact their specialty choice in the future. However, 46.5% were not sure about this statement, as shown in Table 4.

### 3.5. Medical Artificial Intelligence Readiness Scale for Medical Students (MAIRS-MS)

Table 5 shows the students’ responses to the medical AI readiness scale. Regarding cognitive factors, a small percentage of the students agreed that they could define the basics of data science (24.8%), statistics (35.3%), and AI (20.3%). Only 14.7% agreed that they could explain the process of training AI systems. Approximately one-third of the students agreed that they could differentiate the functions and features of AI-related tools and applications and could express the importance of data collection, analysis, evaluation, and safety for the development of AI in healthcare. Additionally, 23.6% of the students agreed that they could properly analyze the data obtained through AI in healthcare, and 28.7% could organize workflows compatible with AI.

Regarding the ability factor, 45.3% of the students agreed that they can combine AI-based information with their professional knowledge, 39.2% can use AI technologies effectively in healthcare delivery, and 44.6% can apply AI as intended.

Regarding the vision factor, 20.8% agreed they could explain the limitations of AI technology, and 36.7% could explain its strengths and weaknesses. Moreover, 30.2% agreed that they could foresee the opportunities and threats that AI technology can create. Regarding the ethics factor, 37.6% agreed that they could use health data in accordance with legal and ethical norms, and 35% agreed to follow legal regulations regarding the use of AI technologies in healthcare. Furthermore, 40.6% agreed that they could conduct their research using ethical principles while using AI technologies.

### 3.6. Total Scores of the Students’ Answers Regarding the Medical Artificial Intelligence Readiness

In addition, the total scores for medical artificial intelligence readiness with its four factors among the medical students are described in Table 6. The total scores ranged from 22 to 110 out of 110, with a median (IQR) of 70 (20). Regarding cognition and ability factors, each factor’s scores ranged from 8 to 40 out of 40, with a median (IQR) of 24 (11) and 27 (8), respectively. Each score of vision and ethics factors ranged from 3 to 15 out of 15, with a median (IQR) of 9 (4) and 12 (3), respectively.

### 3.7. Differences in the MAIRS-MS Scores in Relation to the Sociodemographic and Educational Characteristics

Table 7 shows the significant factors associated with the total MAIR-MS score. Compared with males, females had significantly higher MAIR-MS scores (*p* < 0.001), with a median (IQR) of 72 (21) versus 67 (20) in males. Students in the central region had significantly higher median (IQR) MAIR-MS scores (76 (18)) than those in the western (69 (22)), eastern (72.5 (23)), southern (66 (16)), and northern regions (54 (42)) (*p* < 0.001). Students in the fourth, fifth, and sixth years had significantly higher median (IQR) MAIR-MS scores (72 (19)) than those in the first, second, and third years (69 (31)) (*p* = 0.011).

Additionally, advanced English proficiency and computer technology were significant factors for higher MAIR scores (*p* < 0.001). Moreover, being educated in ethics, data science, or AI was a significant factor for higher MAIR-MS scores (*p* < 0.001). Compared with those with intermediate and basic proficiencies, students with advanced English proficiency had significantly higher median (IQR) MAIR scores (76 (17) vs. 67 (22) and 66 (22)). Additionally, students with advanced computer technology skills had significantly higher median (IQR) MAIR-MS scores than those with intermediate and basic skills (79 (20) vs. 73 (16) and 62 (28)). Furthermore, students who received education in ethics, data science, or AI had significantly higher median (IQR) MAIR-MS scores than those who did not: 72 (18) vs. 62.5 (38) for ethics, 73 (18) vs. 66 (31) for data science, and 78 (23) vs. 69 (20) for AI, respectively. Students with AI training had significantly higher median (IQR) MAIR-MS scores than those who did not (76 (20) vs. 67 (21), *p* < 0.001). Moreover, students who used AI applications had significantly higher median (IQR) MAIR-MS scores than those who did not (71 (21) vs. 59 (47), *p* < 0.001).

### 3.8. Linear Regression Analysis of the Total MAIR-MS Scores

Table 8 illustrates that 32.1% of this study’s results could truly and reliably reflect the influence of gender (*p* = 0.001), area of residency (*p* = 0.001), English proficiency (*p* = 0.025), computer technology (*p* < 0.001), ethics education (*p* = 0.002), data science education (*p* = 0.001), AI training (*p* < 0.001), and AI application use (*p* = 0.001) on MAIR scores by applying linear regression analysis. Gender (B = 0.126, 95% CI: 1.987–7.7308), area of residence (B = −0.125, 95% CI: −3.147 to −0.850), and English proficiency (B = 0.085, 95% CI: 0.332–4.988) are significant predictors of MAIR scores. Additionally, proficiency in computer technology (B = 0.242, 95% CI: 4.223–8.134), ethics education (B = 0.136, 95% CI: 2.120–9.368), data sciences education (B = 0.148, 95% CI: 2.464–9.128), AI training (B = 0.226, 95% CI: 5.731–11.790), and the use of AI applications (B = 0.120, 95% CI: 3.494–13.850) are also significant factors influencing MAIR scores.

## 4. Discussion

This study evaluated the readiness of medical students in Saudi Arabia to embrace artificial intelligence technologies in healthcare using the Medical Artificial Intelligence Readiness Scale for Medical Students (MAIRS-MS). The findings provide valuable insights into the current state of AI education and training among medical students, their cognitive and practical understanding of AI, and the factors that influence AI readiness. Overall, the results indicate moderate AI readiness among the respondents, with notable differences based on gender, region, educational level, and prior training in AI.

The limited exposure to AI education among Saudi medical students echoes findings from a large-scale international survey performed on medical, dental, and veterinary students, as well as another study performed on Arab medical students, where AI education was limited [29,30]. The current study reported that only 14.5% of the students received AI education as part of their curriculum. These findings suggest that AI remains a peripheral topic in many medical school curricula despite its increasing importance in healthcare [31]. Conversely, this situation raises important questions about the optimal timing for integrating AI education into undergraduate medical curricula and, if so, what the content and the most effective educational strategies would be [32,33]. Furthermore, only 34.3% of the students in the present study had received extracurricular AI training, often through self-study, whereas less than 5% had attended hands-on training. This finding is consistent with a previous study that reported that only 9.8% of students had taken AI courses [34]. This further underscores the urgent need for formal AI training in medical education.

A striking finding of this study is the high percentage of medical students who reported using AI applications (93.2%). This suggests that AI is already playing an integral role in the academic lives of Saudi medical students, even though formal AI education remains limited. There is widespread use of AI applications, such as ChatGPT (60.4%), Grammarly (51%), and Poe (40.3%), all of which are designed to assist with natural language processing, writing, and improving communication. The data also revealed that querying medical knowledge (68.2%) and medical research (55.2%) were the most common objectives for utilizing AI applications. These findings are consistent with global trends in education, where AI tools are being increasingly used to assist with learning, writing, and problem-solving [35,36,37]. However, a systematic review suggested that further research with robust validation is necessary to determine the most effective AI tools for medical education [38].

This study offers insights into medical students’ specialty preferences and the emerging impact of AI on career decision-making. Among our participants, 66.25% had chosen their future specialty, with surgery being the most preferred (18.7%), followed by internal medicine (9.4%). This trend contrasts with a recent nationwide survey, where pediatrics (11%) ranked as the most preferred specialty, tied with internal medicine (11%), while general surgery (9%) and neurosurgery (8%) followed in popularity [39]. Notably, specialties like ophthalmology (6%) and family medicine (5%) were also represented in the national data as well as this cohort. Furthermore, a national study revealed that 6.8% of medical students were uncertain about how AI would impact their career paths [40]. While approximately one-third of participants had not decided on their future career yet, 46.5% of medical students were unsure about whether AI advancements would influence their choice of specialty, indicating that while AI is recognized as important, many students may not yet fully understand its practical implications for their future careers. This uncertainty highlights the need for targeted interventions that not only teach AI concepts but also demonstrate their relevance across various medical specialties [41,42].

One of the most significant findings of this study is the association of demographic and educational factors with AI readiness. Compared with their male counterparts, female medical students presented higher AI readiness scores, whereas another national study in Saudi Arabia revealed that male medical students had more positive attitudes toward AI [43]. This finding may suggest that women, particularly in the context of Saudi Arabia, are more open to adopting emerging technologies, potentially because of increasing opportunities and support for women in Science, Technology, Engineering, and Mathematics (STEM) fields [44,45]. The study also revealed regional differences in AI readiness, with students from the central region scoring significantly higher than those from other regions. This could be attributed to differences in access to advanced educational resources and technology across the country, particularly in areas such as Riyadh, which includes at least seven medical colleges.

Educational factors, such as proficiency in English and computer technology, were also strong predictors of AI readiness. Students with advanced English and computer skills scored significantly higher on the MAIRS-MS, which aligns with the technical nature of AI systems, which often require proficiency in these areas. Additionally, students who received education in ethics, data science, or AI scored higher on the readiness scale, which supports the notion that integrating AI education could improve students’ understanding of AI and their capacity to effectively apply it [46]. Moreover, these findings emphasize the need for comprehensive training that addresses not only AI concepts but also their ethical and legal implications in healthcare. This is particularly important given the ethical challenges associated with AI in patient care, such as data privacy, algorithmic bias, and accountability in decision-making, as highlighted in the literature [47,48].

The relevance of this study lies in its potential to drive essential policy reforms in Saudi medical education, aligning with the broader objectives of Vision 2030, which emphasizes technology-driven advancements [49]. To effectively integrate AI training into medical education, institutions should adopt a multi-faceted approach that addresses curriculum design, faculty development, and resource allocation [50]. First, medical schools should establish standardized AI education frameworks that outline core competencies, including fundamental concepts of AI, machine learning, data ethics, and practical applications in clinical settings [51]. Second, collaborations with international academic institutions and industry leaders can provide access to best practices and cutting-edge resources and enrich faculty experience. Additionally, curriculum design should incorporate hands-on learning through simulation-based training, interactive workshops, and exposure to AI tools commonly used in healthcare, such as diagnostic algorithms and decision-support systems.

This study provides valuable insights and includes medical students from all regions of the country, ensuring a diverse representation. However, it is not without limitations. First, the sampling technique may affect the generalizability of the findings to all medical students in Saudi Arabia. Future studies should aim to include a more diverse sample, incorporating students from various academic levels and institutions (public and private) across the country. Additionally, the study relied on self-reported data, which are inherently subject to several limitations that may affect the validity and reliability of the findings, including response bias, recall bias, and measurement bias. Combining self-reported insights with objective AI assessment or institutional data could provide a more accurate measure of AI readiness. Another limitation is the cross-sectional nature of the study, which captures students’ readiness at a single point in time. Longitudinal studies are needed to track changes in AI readiness over time, particularly as AI education becomes more integrated into medical curricula.

While the current study focuses primarily on the readiness and attitudes of medical students toward AI in general, future studies could incorporate specific questions addressing how students perceive and differentiate between AI in medical contexts and Generative AI. Furthermore, research should explore the impact of different AI educational interventions on medical students’ readiness and confidence. Experimental studies that test the effectiveness of various AI training programs, such as online courses, workshops, and AI simulation tools, could provide insights into the best approaches for enhancing AI readiness.

## 5. Conclusions

This study highlights the moderate level of AI readiness among medical students in Saudi Arabia and identifies key factors that influence AI readiness, such as gender, region, and educational background. The findings underscore the need for comprehensive AI education within medical curricula and the importance of equipping future healthcare professionals with the knowledge and skills required to effectively use AI technologies in clinical practice. As Saudi Arabia continues to embrace AI as part of its Vision 2030 initiative, integrating AI education into medical training will be essential for ensuring that the country’s healthcare system remains at the forefront of innovation.

## Figures and Tables

**Table 1:** Sociodemographic characteristics of the medical students (n = 572).

Parameters	Category	Number	Percentage
Gender	Male	272	47.6
	Female	300	52.4
Nationality	Saudi	566	99.0
	Non-Saudi	6	1.0
Area of residence	Western Region	250	43.7
	Central Region	149	26.0
	Eastern Region	80	14.0
	Southern Region	56	9.8
	Northern Region	37	6.5
Medical College	Government	557	97.4
	Private	15	2.6
Medical school year	1st	61	10.7
	2nd	62	10.8
	3rd	107	18.7
	4th	86	15.0
	5th	175	30.6
	6th (Internship)	81	14.2

**Table 2:** Proficiency levels, AI education and training among medical students (n = 572).

Parameters	Category	Number	Percentage
English proficiency	Basic	35	6.1
	Intermediate	313	54.7
	Advanced	224	39.2
Computer technology proficiency	Basic	194	33.9
	Intermediate	266	46.5
	Advanced	112	19.6
Curricular Ethics education	Yes	428	74.8
	No	144	25.2
Curricular Data science education	Yes	385	67.3
	No	187	32.7
Curricular AI education	Yes	83	14.5
	No	489	85.5
Extracurricular AI training	Yes	196	34.3
	No	376	65.7
AI training method(n = 196)	Self-study	111	56.6
	Online training	61	31.1
	Certificate Program	15	7.7
	Hands-on training	9	4.6

**Table 3:** Usage of AI Applications and their objectives among medical students (n = 572).

Parameters	Category	Number	Percentage
AI application use	Yes	533	93.2
	No	39	6.8
AI applications (n = 533)	ChatGPT	322	60.4
	Grammarly	272	51.0
	Poe	215	40.3
	Deep AI	28	5.3
	Copilot	34	6.4
	Other	21	3.9
Objective using AI (n = 569)	Querying medical knowledge	388	68.2
	Medical research	314	55.2
	Explanation of pathologies	280	49.2
	Seminar/presentation	197	34.6
	Diagnostic support	96	16.9
	Therapy suggestions	93	16.3
	Reading radiological images	27	4.7
	Other	14	2.5

**Table 4:** Intended specialties among medical students and the impact of AI advancements (n = 572).

Parameters	Category	Number	Percentage
Intended specialty	Surgery	107	18.7
	Internal medicine	54	9.4
	Family medicine	39	6.8
	Ophthalmology	32	5.6
	Neurology	27	4.7
	Emergency medicine	27	4.7
	Obstetrics and Gynecology	16	2.8
	Pediatrics	15	2.6
	Psychiatry	15	2.6
	Dermatology	13	2.3
	Radiology	12	2.1
	Anesthesia	11	1.9
	Urology	3	0.5
	Pathology	2	0.3
	Orthopedic	2	0.3
	Radiation oncology	1	0.2
	Otolaryngology	1	0.2
	Forensic medicine	1	0.2
	Genetics	1	0.2
Impact of AI advancement on specialty choice	Yes	163	28.5
	No	143	25.0
	Not sure	266	46.5

**Table 5:** Medical Artificial Intelligence Readiness Scale for Medical Students (n = 572).

Factors	Strongly Agree	Agree	Neutral	Disagree	Strongly Disagree
	Number (Percentage)				
**Cognition**					
I can define the basic concepts of data science.	33 (5.8)	142 (24.8)	185 (32.3)	135 (23.6)	77 (13.5)
I can define the basic concepts of statistics.	38 (6.6)	202 (35.3)	145 (25.3)	119 (20.8)	68 (11.9)
I can explain how AI systems are trained.	22 (3.8)	84 (14.7)	161 (28.1)	177 (30.9)	128 (22.4)
I can define the basic concepts and terminology of AI.	25 (4.4)	116 (20.3)	170 (29.7)	155 (27.1)	106 (18.5)
I can properly analyze the data obtained by AI in healthcare.	33 (5.8)	135 (23.6)	166 (29.0)	146 (25.5)	92 (16.1)
I can differentiate the functions and features of AI-related tools and applications.	34 (5.9)	173 (30.2)	161 (28.1)	120 (21.0)	84 (14.7)
I can organize workflows compatible with AI.	38 (6.6)	164 (28.7)	181 (31.6)	111 (19.4)	78 (13.6)
I can express the importance of data collection, analysis, evaluation, and safety for the development of AI in healthcare.	52 (9.1)	166 (29.0)	173 (30.2)	108 (18.9)	73 (12.8)
**Ability**					
I can harness AI-based information combined with my professional knowledge.	50 (8.7)	259 (45.3)	137 (24.0)	68 (11.9)	58 (10.1)
I can use AI technologies effectively and efficiently in healthcare delivery.	47 (8.2)	224 (39.2)	177 (30.9)	84 (14.7)	40 (7.0)
I can use AI applications in accordance with their purpose.	81 (14.2)	255 (44.6)	141 (24.7)	52 (9.1)	43 (7.5)
I can access, evaluate, use, share, and create new knowledge using information and communication technologies.	59 (10.3)	206 (36.0)	188 (32.9)	61 (10.7)	58 (10.1)
I can explain how AI applications offer a solution to an appropriate problem in healthcare.	41 (7.2)	156 (27.3)	211 (36.9)	109 (19.1)	55 (9.6)
I find AI valuable for education, service, and research purposes.	131 (22.9)	217 (37.9)	117 (20.5)	55 (9.6)	52 (9.1)
I can explain the applications of AI in healthcare services to the patient.	45 (7.9)	173 (30.2)	201 (35.1)	101 (17.7)	52 (9.1)
I can choose the proper application for AI to problems encountered in healthcare.	48 (8.4)	174 (30.4)	194 (33.9)	104 (18.2)	52 (9.1)
**Vision**					
I can explain the limitations of AI technology.	37 (6.5)	119 (20.8)	182 (31.8)	147 (25.7)	87 (15.2)
I can explain the strengths and weaknesses of AI technology.	51 (8.9)	210 (36.7)	155 (27.1)	93 (16.3)	63 (11.0)
I can foresee the opportunities and threats that AI technology can create.	44 (7.7)	173 (30.2)	177 (30.9)	112 (19.6)	66 (11.5)
**Ethics**					
I can use health data in accordance with legal and ethical norms.	115 (20.1)	215 (37.6)	135 (23.6)	56 (9.8)	51 (8.9)
I can conduct myself under ethical principles while using AI technologies.	117 (20.5)	232 (40.6)	138 (24.1)	57 (10.0)	28 (4.9)
I can follow the legal regulations regarding the use of AI technologies in healthcare.	119 (20.8)	200 (35.0)	150 (26.2)	51 (8.9)	52 (9.1)

AI: Artificial Intelligence.

**Table 6:** Total scores of the students’ answers regarding the Medical Artificial Intelligence Readiness Scale (n = 572).

The total score of the cognition factor	Mean (SD)	22.69 (7.369)
	Median (IQR)	24 (11)
	Min–Max	8–40
The total score of the ability factor	Mean (SD)	26.12 (7.302)
	Median (IQR)	27 (8)
	Min-Max	8–40
The total score of the vision factor	Mean (SD)	8.97 (2.996)
	Median (IQR)	9 (4)
	Min–Max	3–15
The total score of the ethics factor	Mean (SD)	10.61 (3.268)
	Median (IQR)	12 (3)
	Min–Max	3–15
The total score of the Medical Artificial Intelligence Readiness	Mean (SD)	68.39 (18.316)
	Median (IQR)	70 (20)
	Min–Max	22–110

**Table 7:** Association of the students’ characteristics with total MAIR-MS scores.

Parameters	Category	Median (IQR)	*p* Value
Age (Years)	≤21 (n = 237)	69 (29)	0.74
	>21 (n = 330)	72 (19)	
Gender	Male (n = 272)	67 (20)	<0.001
	Female (n = 300)	72 (21)	
Nationality	Saudi (n = 566)	70 (20)	0.579
	Non-Saudi (n = 6)	76 (34)	
Area of residence	Western Region (n = 250)	69 (22)	<0.001
	Central Region (n = 149)	76 (18)	
	Eastern Region (n = 80)	72.5 (23)	
	Southern Region (n = 56)	66 (16)	
	Northern Region (n = 37)	54 (42)	
Medical College	Governmental (n = 557)	70 (20)	0.113
	Private (n = 15)	62 (62)	
Medical school year	1st, 2nd, and 3rd (n = 230)	69 (31)	0.011
	4th, 5th, and 6th (n = 342)	72 (19)	
English proficiency	Basic (n = 35)	66 (22)	<0.001
	Intermediate (n = 313)	67 (22)	
	Advanced (n = 224)	76 (17)	
Computer technology	Basic (n = 194)	62 (28)	<0.001
	Intermediate (n = 266)	73 (16)	
	Advanced (n = 112)	79 (20)	
Ethics education	Yes (n = 428)	72 (18)	<0.001
	No (n = 144)	62.5 (38)	
Data science education	Yes (n = 385)	73 (18)	<0.001
	No (n = 187)	66 (31)	
AI education	Yes (n = 83)	78 (23)	<0.001
	No (n = 489)	69 (20)	
AI training	Yes (n = 196)	76 (20)	<0.001
	No (n = 376)	67 (21)	
AI application use	Yes (n = 533)	71 (21)	<0.001
	No (n = 39)	59 (47)	
AI advancement and specialty choice	Yes (n = 163)	72 (20)	0.072
	No (n = 143)	71 (18)	

AI: Artificial Intelligence, IQR: Interquartile Range.

**Table 8:** Linear regression analysis of the total MAIR-MS scores.

Factors	Unstandardized Coefficients B	95% CI	*p*-Value
Age	0.065	[−1.297–6.130]	0.202
Gender	0.126	[1.987–7.7308]	0.001
Nationality	−0.043	[−20.348−4.834]	0.227
Area of residence	−0.125	[−3.147–−0.850]	0.001
Medical College	−0.053	[−14.159–2.126]	0.147
Medical school year	−0.062	[−6.397–1.750]	0.263
English proficiency	0.085	[0.332–4.988]	0.025
Computer technology	0.242	[4.223–8.134]	<0.001
Ethics education	0.136	[2.120–9.368]	0.002
Data sciences education	0.148	[2.464–9.128]	0.001
AI education	−0.017	[−4.857–3.109]	0.667
AI training	0.226	[5.731–11.790]	<0.001
AI application use	0.120	[3.494–13.850]	0.001
AI and specialty choice	0.024	[−1.070–2.149]	0.511

CI: Confidence Interval.

## Data Availability

All data used in the study are available to interested researchers upon request from the corresponding author after approval from the Institutional Review Board at KAAUH, PNU (contact irb@kaauh.edu.sa).
